# Supplementary material for: Association between Proton Pump Inhibitor Use and Risk of Hepatocellular Carcinoma: A Korean Nationally Representative Cohort Study
Source: J Clin Med. 2022 May 19;11(10):2865. doi: 10.3390/jcm11102865 (PMC9146713; doi:10.3390/jcm11102865)
Supplement: Supplementary file 1 [file jcm-11-02865-s001.zip › jcm-1712900-supplementary.pdf]

Supplementary Table S1. Association of PPI use with risk of liver cancer among various risk groups.

	Non-User	First tertile	Second tertile	Third tertile	P for trend
Participant, n	341,734	24,344	18,666	21,313	
Overall HCC					
Event (%)	1,215 (0.4)	95 (0.4)	75 (0.4)	81 (0.4)	
Person-year	2,334,893	166,230	127,059	144,325	
Crude rate/10,000 PY	5.2	5.7	5.9	5.6	
HR (95% CI)	1.00 (reference)	1.10 (0.89-1.35)	1.13 (0.90-1.43)	1.08 (0.86-1.35)	0.556
aHR (95% CI)[a]	1.00 (reference)	1.11 (0.90-1.36)	1.09 (0.87-1.38)	0.96 (0.77-1.20)	0.665
aHR (95% CI)[b]	1.00 (reference)	1.07 (0.86-1.31)	1.05 (0.83-1.33)	0.91 (0.72-1.14)	0.731
cirhossis-associated HCC					
Event (%)	236 (0.1)	16 (0.1)	12 (0.1)	18 (0.1)	
Person-year	2,330,710	165,845	126,794	143,926	
Crude rate/10,000 PY	1.0	1.0	0.9	1.3	
HR (95% CI)	1.00 (reference)	0.95 (0.57-1.58)	0.93 (0.52-1.67)	1.23 (0.76-1.99)	0.832
aHR (95% CI)[a]	1.00 (reference)	0.96 (0.58-1.59)	0.90 (0.51-1.61)	1.10 (0.68-1.78)	0.954
aHR (95% CI)[b]	1.00 (reference)	0.96 (0.57-1.59)	0.90 (0.50-1.61)	1.08 (0.66-1.75)	0.966
Non-cirrhosis-associated HCC					
Event (%)	979 (0.3)	79 (0.3)	63 (0.3)	63 (0.3)	
Person-year	2,335,303	166,256	127,084	144,362	
Crude rate/10,000 PY	4.2	4.8	5.0	4.4	
HR (95% CI)	1.00 (reference)	1.13 (0.90-1.43)	1.18 (0.92-1.53)	1.04 (0.81-1.34)	0.446
aHR (95% CI)[a]	1.00 (reference)	1.14 (0.91-1.44)	1.14 (0.88-1.47)	0.93 (0.72-1.20)	0.446
aHR (95% CI)[b]	1.00 (reference)	1.09 (0.87-1.37)	1.09 (0.84-1.40)	0.87 (0.67-1.13)	0.527

HR calculated using the Cox proportional hazards regression.

[a]adjusted for age and sex.

[b]adjusted for age, sex, household income, body mass index, systolic blood pressure, fasting serum glucose, smoking, alcohol consumption, exercise frequency, and Charlson comorbidity index.

Acronyms: DDD, defined daily dose; HCC, hepatocellular carcinoma; PY, person-year; HR, hazard ratio; aHR, adjusted hazard ratio; CI, confidence interval.

Supplementary Table S2. Sensitivity analysis on association of proton pump inhibitor use with risk of HCC after excluding events occurred in specified latent periods.

	**None**	**Low (0 ≤ PPI < 30 DDDs)**	**Intermediate (30 ≤ PPI < 60 DDDs)**	**High (≥60 DDDs)**	**P for trend**
Overall HCC					
1-year washed out	1.00 (reference)	1.04 (0.86-1.26)	0.94 (0.70-1.30)	0.89 (0.63-1.24)	0.828
2-year washed out	1.00 (reference)	1.02 (0.83-1.26)	0.83 (0.59-1.17)	0.70 (0.47-1.05)	0.248
3-year washed out	1.00 (reference)	1.02 (0.81-1.29)	0.79 (0.53-1.16)	0.69 (0.44-1.09)	0.268
Cirrhosis-associated HCC					
1-year washed out	1.00 (reference)	0.98 (0.63-1.51)	0.83 (0.41-1.69)	1.37 (0.74-2.53)	0.717
2-year washed out	1.00 (reference)	0.91 (0.57-1.47)	0.71 (0.31-1.59)	0.84 (0.37-1.90)	0.814
3-year washed out	1.00 (reference)	0.86 (0.50-1.46)	0.57 (0.21-1.53)	0.68 (0.25-1.84)	0.572
Non-cirrhosis-associated HCC					
1-year washed out	1.00 (reference)	1.06 (0.86-1.30)	0.97 (0.70-1.35)	0.77 (0.51-1.14)	0.546
2-year washed out	1.00 (reference)	1.05 (0.84-1.32)	0.86 (0.59-1.25)	0.66 (0.41-1.06)	0.282
3-year washed out	1.00 (reference)	1.07 (0.83-1.38)	0.85 (0.55-1.30)	0.70 (0.42-1.16)	0.416

Data are adjusted hazard ratio calculated using the Cox proportional hazards regression after adjustments for age, sex, household income, body mass index, systolic blood pressure, fasting serum glucose, smoking, alcohol consumption, exercise frequency, and Charlson comorbidity index.

Acronyms: DDDs, defined daily dose; HCC, hepatocellular carcinoma; HR, hazard ratio; aHR, adjusted hazard ratio; CI, confidence interval.

Supplementary Table S3. Stratified analyses of association of proton pump inhibitor use with risk of overall hepatocellular carcinoma.

			aHR (95% CI) by the defined daily dose of PPI use during 4 years					
	**Total**	**Event**	**None**	**Low (0 ≤ PPI < 30 DDDs)**	**Intermediate (30 ≤ PPI < 60 DDDs)**	**High (≥60 DDDs)**	**P for trend**	**P for interaction**
Age								
<60 years	295,141	894	1.00 (reference)	0.99 (0.79-1.24)	0.98 (0.69-1.39)	0.77 (0.49-1.21)	0.726	0.445
≥60 years	110,916	572	1.00 (reference)	1.21 (0.94-1.57)	0.97 (0.64-1.47)	1.07 (0.70-1.63)	0.519	
Sex								
Men	215,550	1,116	1.00 (reference)	1.04 (0.86-1.27)	1.00 (0.74-1.35)	0.84 (0.58-1.20)	0.754	0.695
Women	190,507	350	1.00 (reference)	1.20 (0.86-1.67)	0.88 (0.48-1.61)	1.16 (0.63-2.12)	0.674	
Household income								
Low half	150,613	609	1.00 (reference)	1.32 (1.03-1.70)	1.43 (1.10-2.05)	0.96 (0.60-1.54)	0.046	0.013
Upper half	255,444	857	1.00 (reference)	0.93 (0.74-1.17)	0.68 (0.45-1.03)	0.86 (0.57-1.30)	0.263	
Obesity (≥25 kg/m^2)								
Yes	140,057	512	1.00 (reference)	1.00 (0.75-1.34)	0.76 (0.46-1.26)	0.63 (0.35-1.15)	0.346	0.385
No	266,000	954	1.00 (reference)	1.13 (0.91-1.39)	1.10 (0.80-1.52)	1.06 (0.74-1.53)	0.675	
Charlson Comorbidity Index								
0	179,113	554	1.00 (reference)	1.08 (0.77-1.52)	1.26 (0.75-2.10)	1.01 (0.48-2.13)	0.819	0.702
≥1	226,944	912	1.00 (reference)	1.08 (0.89-1.31)	0.90 (0.65-1.23)	0.88 (0.63-1.24)	0.631	
Smoking								
Yes	116,169	662	1.00 (reference)	1.06 (0.82-1.36)	0.99 (0.66-1.46)	1.12 (0.75-1.70)	0.927	0.560
No	289,888	804	1.00 (reference)	1.10 (0.88-1.38)	0.97 (0.67-1.40)	0.72 (0.45-1.15)	0.423	
Alcohol consumption week/month								
Never	243,474	680	1.00 (reference)	1.32 (1.05-1.66)	0.91 (0.60-1.37)	1.02 (0.67-1.56)	0.108	0.089
1week or over	162,583	786	1.00 (reference)	0.88 (0.68-1.13)	1.03 (0.72-1.47)	0.79 (0.50-1.25)	0.576	
Physical activity, time/week								
Never	213,675	753	1.00 (reference)	1.21 (0.96-1.51)	1.08 (0.75-1.54)	1.01 (0.68-1.51)	0.434	0.307
1 time or over	192,382	713	1.00 (reference)	0.95 (0.73-1.22)	0.87 (0.58-1.30)	0.78 (0.48-1.27)	0.674	
CLD								
Yes	83,359	490	1.00 (reference)	0.85 (0.64-1.12)	1.16 (0.81-1.70)	0.93 (0.61-1.41)	0.497	0.049
No	322,698	976	1.00 (reference)	1.16 (0.94-1.44)	0.70 (0.46-1.06)	0.73 (0.46-1.16)	0.077	
GERD								
Yes	86,134	253	1.00 (reference)	1.03 (0.76-1.38)	0.86 (0.57-1.32)	0.90 (0.59-1.37)	0.850	0.249

No	319,923	1,213	1.00 (reference)	1.27 (1.02-1.58)	1.32 (0.91-1.89)	1.27 (0.78-2.09)	0.070	
ASP								
Yes	30,368	146	1.00 (reference)	0.70 (0.37-1.34)	1.66 (0.70-3.09)	0.71 (0.26-1.92)	0.207	0.123
No	375,689	1,320	1.00 (reference)	1.12 (0.94-1.34)	0.89 (0.66-1.20)	0.93 (0.67-1.28)	0.439	
NSA								
Yes	337,985	1,149	1.00 (reference)	1.13 (0.94-1.35)	0.92 (0.68-1.25)	0.91 (0.65-1.28)	0.497	0.566
No	68,072	317	1.00 (reference)	0.90 (0.58-1.41)	1.29 (0.73-2.25)	0.88 (0.41-1.87)	0.762	

The estimates were based on fully adjusted models: age, sex, household income, body mass index, systolic blood pressure, fasting serum glucose, smoking, alcohol consumption, exercise frequency, and Charlson comorbidity index.

Acronyms: aHR, adjusted hazard ratio; CI, confidence interval; DDD, defined daily dose; CLD, Chronic Liver Disease; GERD, Gastroesophageal Reflux Disease; ASP, Aspirin; NSA, Non-Steroidal anti-inflammatory drugs.
